# Women's Beliefs about Male Circumcision, HIV Prevention, and Sexual Behaviors in Kisumu, Kenya

**DOI:** 10.1371/journal.pone.0097748

**Published:** 2014-05-20

**Authors:** Thomas H. Riess, Maryline M. Achieng', Robert C. Bailey

**Affiliations:** 1 School of Public Health, University of Illinois at Chicago, Chicago, Illinois, United States of America; 2 Liverpool VCT, Care and Treatment, Research and Policy Department, Nairobi, Kenya; Johns Hopkins University Bloomberg School of Public Health, United States of America

## Abstract

It is important to understand how women's sexual practices may be influenced by male circumcision (MC) as an HIV prevention effort. Women's beliefs about MC and sexual behaviour will likely influence the scale-up and uptake of medical MC. We conducted qualitative interviews with 30 sexually active women in Kisumu, Kenya. Women discussed MC related to perceived health benefits, condom use, sexual behaviour, knowledge of susceptibility to HIV and sexually transmitted infections (STIs), circumcision preference, and influence on circumcision uptake. Respondents had a good understanding of the partial protection of MC for acquisition of HIV for men. Women perceived circumcised men as cleaner, carrying fewer diseases, and taking more time to reach ejaculation. Male's circumcision status is a salient factor for women's sexual decision making, including partner choice, and condom use. It will be important that educational information affirms that MC provides only partial protection against female to male transmission of HIV and some STIs; that other HIV and STI prevention methods such as condoms need to be used in conjunction with MC; that MC does not preclude a man from having HIV; and that couples should develop plans for not having sex while the man is healing.

## Introduction

The role of women's sexual behavior as it relates to men's circumcision status is an important component of HIV prevention that requires investigation. Since women make up 50% of persons living with HIV globally, and women's share of infections is increasing, it is important to understand how male circumcision (MC) for HIV and sexually transmitted infection (STI) prevention can impact women's sexual practices [1]. While the results of three randomized controlled trials have shown that MC reduces female to male transmission of HIV by approximately 60% during vaginal intercourse, there are also direct benefits for women, including reducing the risk of STIs, cervical cancer, and possibly HIV [2]–[6].

The strength of MC's protective effect for women depends in part on the percent of men that are circumcised. Women would benefit from MC in the long-term at the population level through herd immunity, since having fewer HIV infected men as a result of circumcision, will reduce the chances of women becoming infected by men [7], [8]. But significant population level changes in HIV incidence among women would take several years to occur [9]. In the short-term a woman's risk for HIV could be significantly reduced if circumcision prevents her male partner from becoming infected with HIV [10].

In addition to providing partial protection against HIV, data have shown that MC reduces the risk of men becoming infected with the human papillomavirus (HPV) by 35%, and herpes simplex virus-2 by 25% [11]–[14]. The risk reduction of HPV transmission due to MC offers women protection against cervical cancer since it reduces the prevalence of oncologic strains of HPV among men [15]–[18]. Female partners of circumcised men are at lower risk of cervical cancer caused by persistent infection with high-risk types of HPV [19]–[21]. The fact that MC confers partial protection against some STIs can serve to deter HIV infection since the presence of STIs are known to increase susceptibility to HIV [22], [23].

In contrast, some public health experts argue that through the promotion of MC women will be made more vulnerable to HIV and STIs because circumcised men may believe that, due to the HIV and STI protection afforded them by MC, they can reduce or eliminate condom use or that they may resume sex before the healing process is complete [24]. This could compromise a woman's ability to negotiate condom use, particularly in contexts where men exert economic and physical power over women [25]. Further, women could be blamed or stigmatized as vectors of HIV and STIs, leading to greater feminization of the HIV epidemic as they are accused of introducing HIV into a relationship [26].

Research exploring the reduction of HIV transmission from circumcised men to women has shown less promising results. A RCT looking at HIV transmission between circumcised and uncircumcised HIV positive men and their female sex partners stopped enrollment after the trial's data safety monitoring board concluded that the study would not likely show a reduction in HIV risk for women [27]. An observational study by Gray et al., which examined HIV transmission from uncircumcised and circumcised HIV positive men to HIV negative female partners, showed a greater HIV incidence in women with uncircumcised partners (13.2/100 person years) versus in those with circumcised partners (5.2/100 person years), but these results were not statistically significant [28]. The results varied depending on HIV viral load, with viral loads of 50,000 copies/ml and above having equal rates of transmission. A clinical trial conducted in seven eastern Africa countries found that female partners of circumcised men had an approximately 41% lower risk of HIV acquisition compared to female partners of uncircumcised men; these results were borderline significant [5].

Women play an important part in influencing male circumcision uptake. Women have been shown to influence and make decisions about whether their sons are circumcised as well as sway their male sexual partner's decision to become circumcised [29], [30]. Acceptability studies have shown that 47–79% of women in Kenya, South Africa, and Botswana favor circumcision for their sexual partners and an even greater number, 62–89%, of women were willing to circumcise their sons [31]. Women can also be a source of information about MC for their male partners, and there is evidence that a woman's preference for a circumcised partner is influencing male interest in circumcision [10], [32]–[34].

As MC is being scaled up in many sub-Saharan African countries, little research has been carried out to investigate women's perceptions of circumcised and uncircumcised men, their influence on MC uptake, and how their sexual behaviors are influenced by MC status. Understanding HIV risk behaviors between women and men in the context of medical male circumcision (MMC) scale-up deserves attention. This paper presents findings pertaining to how women's perceptions of circumcised and uncircumcised men and knowledge of MC risk reduction for HIV and STIs influence their sexual risk behaviors and MC preferences.

## Methods

### Ethics Statement

Ethical approval for our research was obtained from the University of Illinois at Chicago in the United States and Kenyatta National Hospital in Kenya. All staff received training on ethical research procedures and completed an online training course on human subjects protection.

### Study Context

This study was carried out in Kisumu, Kenya, the country's third largest city with a population of approximately 470,000. Nyanza Province, of which Kisumu is the capitol, has the highest HIV prevalence in Kenya at 15.1% [35]. The main ethnic group in Kisumu is the Luo.

Approximately 91% of Kenyan men are circumcised. Luo men do not traditionally circumcise, and it is estimated that 66% of Luo men in Nyanza Province are circumcised, haven risen from 44.8% in 2007 [35], [36]. MC has been promoted in Kisumu since a RCT that examined MC's affect on HIV incidence was concluded in December 2006. Since the end of the RCT, MC has become more widely available in Nyanza Province through public and private health facilities and is being more widely adopted among Luo boys and men. This study was conducted from March to November 2008, as MC for HIV prevention was being scaled up.

### Respondents and Data Collection

We conducted qualitative individual in-depth interviews that targeted women 18 to 35 years old, who were sexually active in the past 12 months, resided in Kisumu District, and were willing to be audio recorded. Respondents were recruited from health clinics, on the street, and at shopping centres using purposive sampling methods [37]. We sought women from the general population in Kisumu in order to obtain a wide variety of respondents. Interview respondents were also obtained through snowball sampling whereby participants were asked to refer women who would be rich sources of information on sexual risk perceptions and behavior related to MC [38]. Potential respondents were screened for eligibility and scheduled for an interview. All respondents received an oral and written explanation of the study's procedures and objectives and each provided signed consent to be interviewed and audio-recorded.

A semi-structured interview guide focused on the respondent's knowledge, experience, and perceptions of MC and HIV prevention guided the interviews. Table 1 provides a partial list of interview questions and probes. Interviews were conducted in Swahili, Dholuo, and English depending on the respondent's language preference. Interviews lasted 50 to 96 minutes. At the conclusion of the interview respondents were administered a 35-item demographic questionnaire. All respondents were reimbursed 150 Kenyan shillings (approximately US$2.25). After completing 30 interviews we reached a point of saturation whereby conducting additional interviews was unlikely to produce new insights.

**Table 1:** Key individual interview guide questions.

• Tell me what you know about male circumcision.
• What are your feelings about male circumcision? (Probes: *What does MC mean to you? Does your ethnic/cultural heritage influence how you think or feel about MC?*)
• What do you know about circumcision's relationship to HIV? (Probe: *How did you learn what you know about circumcision and its relationship to HIV/STIs?*)
• Have your views around circumcision changed since finding out that circumcision reduces the chance of HIV transmission?
• In your own words tell me what are some of the differences between circumcised and uncircumcised men? (Probes: *Desirability, hygiene, religion, greater/lower ability to please partners, perceived risk of HIV and/or STIs, difference of having sex with a circumcised versus an uncircumcised man?*)
• What do you think about sexual partners that are uncircumcised?
• What do you think about sexual partners that are circumcised? (Probes: *Is there a difference in your desire to practice safe sex depending on a man's circumcision status? Do you have a preference for circumcised or uncircumcised men?*)
• How has whether or not a man is circumcised influenced how you thought of him? (Probes: *Sexual desirability, hygiene, religion, ethnic affiliation, greater/lower ability to please partners, reduced perceived risk of HIV and/or STIs.*)
• Is your regular partner circumcised? (Probe: *Has he been circumcised since you have been sexually active with him?*)
• Tell me what sexual practices have changed between you and your partner after he was circumcised. (Probes: *Type of sex, condom use, how long did you wait to have sex after his circumcision?*)
• What influence did you have on him getting circumcised? (Probes: *Did you want him to? Not want him to? Why/why not? What would he have done with/without your support?*)

### Analysis

All audio recordings of interviews were transcribed verbatim in the language of the interview, and then translated into English, if necessary. Transcribed interviews were imported into ATLAS.ti qualitative data analysis software for coding [39].

Codes were developed by the research team from activities, relationships, meanings, context and perspectives that emerged from the interviews using open and axial coding procedures of grounded theory [40]. After coding the interviews they were analyzed using the constant comparative method and an inductive framework in which categories, themes, and patterns emerged from the data [41], [42].

A quality assurance protocol was used in order to monitor the accuracy of verbatim transcription and inter-coder reliability. Fifty percent of the transcripts were checked to verify the accuracy of transcription and twenty percent of interviews were coded by two members of the research team, who coded interviews independently and then met and devised a mutual coding scheme.

## Results

We present findings from individual in-depth interviews with 30 women (N = 30). Table 2 presents respondent demographic characteristics. The mean age of our study respondents was 24.8 years and ranged from 20 to 33 years. A majority of women were from the Luo ethnic group (87%), were not married and not living with a sex partner (84%), had a secondary school education or higher (64%), and earned less than 5,001 Kenyan Shillings (approximately US$67) per month (80%). Respondents accurately identified correct and incorrect routes of HIV transmission and correct and incorrect methods of HIV prevention.

**Table 2:** Demographic characteristics of respondents (N = 30).

Characteristics	N	%
**Age (years)**		
20–23	13	30
24–27	11	50
28–33	6	20
**Marital Status**		
Married living with spouse	4	13
Married not living with spouse	1	3
Not married and not living with sex partner	25	84
**Highest education level completed**		
Did not finish primary school	3	10
Primary school	8	27
Secondary or vocational school	11	37
Beyond secondary school	8	27
**Average monthly income**		
<2,000 Shillings	13	43
2,000–5,000	11	37
5,001–10,000	3	10
>10,000	3	10
**Ethnic Group**		
Luo	26	87
Luhya	2	7
Kisii	2	7

Among the cohort of women 57% reported having one sexual partner and 30% reported having two sexual partners in the past twelve months, 57% have had sex with both uncircumcised and circumcised men, 23% have had sex with circumcised men only, 20% have had sex with uncircumcised men only, and 70% reported that their most recent sex partner was circumcised.

The results of our analysis are presented below as six overlapping themes: 1) perceived benefits of MC, 2) condom use and MC status, 3) sexual behavior and MC status, 4) MC and knowledge of HIV & STI susceptibility, 5) circumcision preferences, and 6) women's influence on circumcision uptake.

### Perceived Benefits of Male Circumcision

Respondents' awareness that MC provides partial protection against HIV and STIs was sometimes interpreted as meaning that circumcised men were less likely to be infected with HIV. Some women also perceived circumcised men as more hygienic, which they described as the penis having no, or less, odor than an uncircumcised penis. For others MC was perceived as allowing men to take longer to ejaculate, which in turn made sex more enjoyable for women. The respondent below described multiple perceived benefits of MC for herself.

***Interviewer***: *And what are your feelings about male circumcision?*

***Respondent*** *: I think it's good. Yeah, I think it's very good. Because, for one it reduces chances of getting HIV. And then I can go on [having sex] for a while instead of just, you're just starting and there he's done.*

***I**: Any other reasons why you think it might be good?*

***R**: Yeah, I think it's also hygienic.*

***I**: Hygienic in what way*

***R**: I hear that there is some stuff, which at times remains on the foreskin, and the kind of dirt, they are smelly. (21 year-old Luo woman)*

Given that most women believed that they don't receive any direct health benefit from MC their views sometimes focused on the sexual experience with a circumcised versus an uncircumcised man or hygiene, and not HIV prevention. One woman described this as more important than the risk reduction of HIV and STIs, which she saw as a benefit for the man.

***I**: … do you think you were going to gain by having him circumcised?*

***R**: … See I had already known that circumcised men are good in bed, that is benefit number one of course, the main benefit. The second benefit, maybe for his own good, … his risk of contracting HIV/AIDS is low. (25 year-old Luo woman)*

### Condom Use and Male Circumcision Status

No respondents reported changes in condom use due to a male sexual partner's circumcision status. Seventy percent of women reported using condoms during their last sexual encounter and 47% said they used condoms in all sexual encounters over the previous 12 months. More than half of respondents (70%) reported that only themselves, or mostly themselves, decided when to use condoms in their sexual relationships. Women who reported not using condoms consistently said that this was due to using other birth control methods, being in a long-term sexual relationships, or because condoms decreased sexual pleasure.

A majority of women reported that a man's circumcision status would not affect their condom use because they were aware that someone could have HIV regardless of circumcision status. As the women below explained:

***I**: … say you know, a guy is circumcised, have you ever thought, well maybe I don't need to use condoms with him because he is at lower risk, and you look at a non-circumcised guy and you're like, oh maybe he's higher risk, so I have to use a condom?*

***R**: No, no, no, no. You know I can't say that circumcised men are protected from STIs. Cause maybe the circumcised man is HIV infected while the uncircumcised man is not. (23 year-old Luo woman)*

Not knowing a man's HIV status was reason enough for some women to disregard circumcision status and encourage condom use.

***R**: … if I don't know their [HIV] status, definitely I have to tell them to use a condom, whether they are circumcised or not. Because you can't judge whether one is HIV positive or HIV negative. (24 year-old Luhya woman)*

No respondents indicated that they would engage in higher-risk sexual activity, including stopping condom use, because a man was circumcised. However, two women said that after learning about MC and its HIV risk reduction properties, they decided it was more important to use a condom with uncircumcised men.

*I think it really counts when you use protection like for the uncircumcised. Okay, for the uncircumcised I might really prefer to use a condom. … it's for the hygienic part. I really do feel that maybe I might be prone to more infections with the man who is uncircumcised. (26 year-old Luo woman)*

### Sexual Behavior and Male Circumcision Status

All but one woman indicated that their sexual behavior would not change because of a man's circumcision status. Consequently there were no reported increases or decreases in the number of sexual partners related to men's circumcision status, nor were there any reports of women engaging in higher-risk sex, such as unprotected anal sex, because a man was circumcised and had a lower risk for contracting HIV or STIs. Respondents talked about circumcision status as an indicator of female sexual satisfaction, time to ejaculation, and differences in male libido, but for the most part did not change their sexual behavior due to these factors.

The one respondent who reported that she would change her sexual behavior between circumcised and uncircumcised men said she would not perform oral sex on an uncircumcised man, but would perform it on a circumcised man. This illustrates that some women do differentiate sexual activity that they will engage in with circumcised and uncircumcised men.

***I**: … So are there things during sex that you would be more willing to do with a circumcised guy that you would not do with a non-circumcised guy?*

***R**: Yes. Like when I look at the dick of uncircumcised person, that skin irritates me, like I can't like even suck it you know. I feel it's dirty.*

***I**: But for a circumcised guy?*

***R**: Yeah, I'll willingly do the stuff [perform oral sex]. (23 year-old Luo woman)*

### Male Circumcision and Knowledge of HIV and STI Susceptibility

Twenty-two women (73%) knew that circumcised men had a lower risk for contracting HIV and STIs. There was not a clear sense among respondents about the percentage of risk reduction that MC provided, with only two respondents knowing that there was approximately a 60% reduction in HIV transmission from women to men and two respondents erroneously stating that MC provided total protection against HIV. Three women did not know about the reduced risk for men contracting HIV and five had heard of such a relationship but did not believe it to be true. Respondents learned about MC's reduced risk of contracting HIV and STIs from husbands, boyfriends, AIDS service organizations, television, radio, church, friends, and teachers.

Respondents' reported that MC reduced the risk of HIV transmission by not allowing “dirt”, “diseases”, or vaginal fluids to exist under the foreskin. Thus with circumcised men, since there is no foreskin, there would be no such hidden dirt and diseases, lowering a woman's risk of contracting HIV and STIs.

***R**: I believe a circumcised man he's clean. And this uncircumcised one he's dirty. … The dirt is now remaining on the foreskin.*

***I**: … so what does it mean to be clean?*

***R**: As when he's circumcised I believe he cannot, he's free from these STIs. And this one who is not circumcised I believe he can contact these STIs very fast. (26 year-old Luo woman)*

Some women said that circumcised men were free from STIs and HIV, equating circumcision status with negative HIV or STI status.

*What I hear about it [male circumcision] is that it's very helpful to men. Like I do hear that men who are circumcised, there is no chances of them to contract HIV or these other STIs, like candidiasis, because the foreskin is not there (29 year-old Luo woman)*

In some cases women's knowledge of MC's protection against HIV and STIs directed their sexual partner selection based on circumcision status, thinking that it would reduce their chance of contracting diseases.

***I**: And say you get some man who is not circumcised, what will you do?*

***R**: You tell him that circumcision is good, a circumcised person has less chances of getting infected with these diseases, these minor diseases.*

***I**: And if he still refuses?*

***R**: If he refuses you just leave him. (27 year-old Luo woman)*

### Circumcision Preferences

Twenty-three (77%) women said they preferred circumcised men, two (6%) women indicated a preference for uncircumcised men, and five (17%) women had no circumcision preference for their sexual partners. Respondent's reasons for preferring circumcised men varied from their being more hygienic, to taking longer to ejaculate, to providing some level of HIV or STI protection for the woman.

***I**: Do you desire circumcised men?*

***R**: Of course a circumcised one (laughs).*

***I**: Why not the uncircumcised one?*

***R**: I don't want diseases. (22 year-old Luo woman)*

A respondent who worked as a sex worker had a preference for circumcised men for her romantic relationships, but when it came to sex for money she preferred uncircumcised men because according to her they ejaculated quicker.

***I**: … do you like circumcised ones or the uncircumcised ones?*

***R**: … let's just say that now I've got a man and I want sex to satisfy my needs. If you want to be satisfied, … you go for the circumcised ones. Now this is just for romantic, for my body's needs. Because my body needs sex, you go for the circumcised. But when you are going to go for money, we go for the uncircumcised ones.*

***I**: Okay, why would you prefer him [uncircumcised]?*

***R**: Cause they are quick. (26 year-old mixed Kisii and Luo woman)*

Health issues besides HIV and STIs were a concern of some women. The woman below justified her preference for circumcised men through a fear of cervical cancer.

***R**: I believe I can't move on with uncircumcised men. …. Because I really fear about cancer, cervical cancer. So I believe, men who are not circumcised I can't be a hundred percent if they're really taking care of the personal hygiene. …*

***I**: So you prefer which?*

***R**: I prefer a man who is circumcised. (24 year-old Luhya woman)*

In some cases women did not describe why they preferred circumcised men but rather described undesirable aspects of uncircumcised men. A woman who had both uncircumcised and circumcised sexual partners was critical towards uncircumcised men because she said that they did not please her as much sexually as circumcised men, who she claimed took longer to ejaculate.

*… no matter how the lubrication is, that foreskin will, I don't know, it moves … and then let me say they don't stay long. … Yeah they didn't stay long when you guys are the uncircumcised. Out of curiosity I did ask how come you don't take long. They say like if that skin is moving it makes them crazy and they release so fast, and I said, okay. And then unlike the circumcised people maybe it's to our advantage, the ladies, maybe it could be not to them but I think to our advantage they'll take long. Like they might make you reach a peak faster than the uncircumcised. (23 year-old Luo woman)*

A minority of women felt that there was no difference between sex with a circumcised and uncircumcised man and therefore had no preference.

*It all depends with a man for example if someone is circumcised and he doesn't know, I mean he is not good in that [sex]. He won't know. When someone is circumcised it doesn't mean that he's now good in bed. And also the one who is uncircumcised, if he is good in bed, he is good in bed. It doesn't matter. (20 year-old Luo woman)*

The two Luo women who preferred uncircumcised men had not had circumcised sexual partners. One reported not understanding, and the other reported not believing the partial protection that MC provides against HIV.

### Women's Influence on Circumcision Uptake

Some women felt they influenced men to get circumcised by talking to them about MC or by insisting that men get circumcised. In some cases it appeared that women were more informed about the benefits of MC than their male partners and they in turn educated men and encouraged them to get circumcised.

*Women who have some knowledge, awareness on male circumcision, are really willing for their men to participate. Like my husband was just circumcised recently. If at all the woman is aware or has undergone some of the awareness and know how male circumcision reduces the risk of HIV, like you know nowadays people really fear HIV. … So most women, who have at least awareness on that, are really encouraging their husbands to be circumcised. (29 year-old Luo woman)*

Five women reported that when they met uncircumcised men with whom they were interested in having a sexual relationship, they insisted that the men get circumcised before they have sex. The respondents who described this situation said they would not have sex with an uncircumcised man. The woman below ended her relationship with a man that she was seeing because he was uncircumcised. She got back together with him five months later, after he was circumcised.

***R**: Actually, me personally, I hate uncircumcised men.*

***I**: Why?*

***R**: I just feel they are dirty and, … this last time, some other guy seduced me, … I didn't know he was uncircumcised. So when we went out a bit for around four months, so it's this day was he was telling me like we go to bed, after finding out that the guy is uncircumcised I just told him it can't work. He should go get circumcised first and come back.*

***I**: So how did he react?*

***R**: Well actually he felt bad, but later he came to understand. That is when he went and got circumcised and we are together now. (25 year-old Luo woman)*

The respondent below believed that it was important to incorporate women into the MC process because she thought that women could be influential in persuading men to get circumcised. She also felt that women should be able to voice their opinion as to whether a man gets circumcised or not, because a man may get circumcised against the wishes of the woman causing discord in a relationship.

*Male circumcision should involve women also, to have a voice and also to learn. Because women are the people who can encourage men to go for circumcision. You know as women, there is a way you can talk to a man to accept something rather than a man coming to you directly, … so the best thing also for women to be involved in the awareness, also in the counselling. (24 year-old Luhya woman)*

## Discussion

This study set out to investigate women's in-depth beliefs about circumcision and how their views of MC are related to their sexual preferences and behaviors. Our results indicate that women in Kisumu, Kenya care about men's circumcision status and it is a salient factor in sexual decision-making, including partner selection and condom use. This indicates that women will likely have a significant influence on acceptability and uptake of MC as it is scaled up in western Kenya and elsewhere in sub-Saharan Africa. Respondents were aware that MC provides men partial protection against HIV, but the benefit that they cited most, improved male hygiene and cleanliness, was a reason to prefer circumcised versus uncircumcised sexual partners. Some women also believed that circumcised men are slower than uncircumcised men to ejaculate, thus giving women greater sexual satisfaction. These results are consistent with findings from studies in Nyanza Province, Kenya, Tanzania and in Uganda where women were asked about their sexual satisfaction after their male partners were circumcised with 37% stating that they were more satisfied after their partner was circumcised versus 2% who were more satisfied when their partners were uncircumcised [34], [43]–[45]. While some women support MC based on their personal experience and beliefs, there may also be the potential for discrimination against uncircumcised men as circumcision programs scale up in sub-Saharan Africa. Program implementers should ensure that MMC promotion campaigns and counselling are clear that studies have shown that MC does not affect male time to ejaculation [46]–[48].

Risk compensation in the face of widespread promotion of MC is a concern [49]. However, higher risk sexual behavior, including decreasing condom use or increasing the number of partners in response to men getting circumcised was not reported by the women in our study. Respondents had a good general understanding of MC's protective affect against HIV and STIs. While only two women knew that MC provided a 60% risk reduction for female to male HIV transmission, there was a general understanding that MC was only partially protective for men and that being circumcised did not indicate that a man is not HIV infected. Indeed, knowledge that MC is only partially protective was reported as a reason why some women did not engage in higher risk sex or alter their condom use. The two women who reported that they would increase their condom usage with uncircumcised men indicate that women may make decisions about condom use based on a man's circumcision status. Respondents whose male partners were circumcised during their relationship (N = 2) did not report engaging in sex before the man had fully healed, which is a legitimate concern since, during present scale-up of MC programs in Kenya, approximately 31% of men have been found to engage in sex before they are fully healed [50], [51].

MC programs afford an opportunity to engage men and women in couples counselling. Including women in pre-circumcision counselling can provide an opportunity to dispel myths about uncircumcised and circumcised men. Learning correct information may shape their sexual behavior in ways that can decrease the risks of HIV and STI transmission. Based on our results, it will be important to include information that MC provides only partial protection against female to male transmission of HIV and some STIs; that other HIV and STI prevention methods such as condoms need to be used along with circumcision; that MC does not preclude a man from having HIV; and that couples should develop plans for not having sex while the man is healing. Since the sexual gratification of both partners is important to the success of MMC programs and some women in this study expressed greater sexual satisfaction with circumcised partners, messaging around women's sexual pleasure may be worthwhile to explore for MMC campaigns in order to promote the idea of women's sexual pleasure as part of MMC.

As MC is promoted and scaled up it has the potential to create new social norms around sexuality and HIV prevention behavior in communities that traditionally do not circumcise. Despite having a general understanding of MC's protective effects our respondents reported varying views about MC. Some women we interviewed perceived a relationship between MC and male sexual performance, libido, hygiene, and incorrectly believed that MC was an indication of reduced probability of being infected by HIV or STIs from circumcised men. Previous research has shown that women who prefer circumcised partners were six times more likely to believe that circumcised men are less likely to be HIV infected [52]. The women we interviewed showed an overwhelming preference for circumcised male sex partners and reported being more concerned about the acquisition of diseases from uncircumcised men. Our interviews with mostly unmarried women suggest that there may be a cultural preference developing for circumcised men as sexual partners, based on proven (male risk reduction for HIV and STIs) and unproven (male hygiene, time to ejaculation, and sexual performance) attributes of circumcision status. It is notable that 70% of the women in our study reported having had sex with a circumcised man in a province with a circumcision rate of around 45% at the time of data collection [36]. These results are similar to those from a survey conducted in western Kenya where 62% of women said that they would prefer circumcised partners [53]. A much lower percent of our respondents (23%) reported having had sex with uncircumcised men only, which could indicate that partner selection based on circumcision status is already occurring.

There are limitations to this study. Since we relied on self-reports it is possible that some respondents could have fabricated answers or not fully disclosed information based on what is socially acceptable, particularly on sensitive topics such as sex and HIV. We did attempt to select respondents who were representative of sexually active women ages 18-35 but given the small sample size and geographic location of our research, our data might not be generalizable to other populations, particularly those where MC is not being promoted as HIV prevention. Our intention has been to gain insights into female perceptions and sexual behaviors related to MC in western Kenya in order to inform and improve programs scaling up MMC for HIV prevention in the region.

Additional research is needed that examines the most effective ways to engage women in maximizing the positive and minimizing the deleterious consequences of MC for themselves and their male sexual partners. Since data was collected for this study the prevalence of MC in Kisumu has risen to 66% so it would be desirable to replicate this study now that a greater percentage of men are circumcised [35]. Our study identifies areas that merit prevention education efforts targeting women and couples. Collecting further data about women's perceptions and experiences related to MC will assist in developing messages around negotiating safe sex and how MC interfaces with other HIV prevention methods, such as condoms. Further, targeting educational messages to women should help to increase acceptance of MC by men for themselves and as lovers, partners, and fathers, making significant impacts on both male and female HIV incidence in high prevalence regions.

## References

[1] UNAIDS (2010) Global report: UNAIDS report on the global AIDS epidemic. Geneva, Switzerland.

[2] Auvert BD, Taljaard E, Lagarde J, Sobngwi-Tambekou J, Sitta R, et al (2005) Randomized, controlled intervention trial of male circumcision for reduction of HIV infection risk: the ANRS 1265 trial. *PloS Medicine* 2(11): e298. 16231970 10.1371/journal.pmed.0020298 PMC1262556

[3] Bailey RC, Moses S, Parker CB, Agot K, Maclean I, et al (2007) Male circumcision for HIV prevention in young men in Kisumu, Kenya: a randomized controlled trial. *The Lancet* 369(9562): 1–15. 10.1016/S0140-6736(07)60312-2 17321310

[4] Gray RH, Kigali G, Serwadda D, Macomb F, Watya S, et al (2007) Male circumcision for HIV prevention in men in Rakai, Uganda: a randomised trial. *The Lancet* 369(9562): 657–666. 10.1016/S0140-6736(07)60313-4 17321311

[5] Baeten JM, Donnell D, Kapiga SH, Ronald A, John-Stewart G, et al (2010) Male circumcision and risk of male-to-female HIV-1 transmission: a multinational prospective study in African HIV-1-serodiscordant couples. *AIDS* 24(5): 737–744. 20042848 10.1097/QAD.0b013e32833616e0 PMC2919808

[6] Hallett TB, Alsallaq RA, Baeten JM, Weiss H, Celum C, et al (2011) Will circumcision provide even more protection from HIV to women and men? New estimates of the population impact of circumcision interventions. *Sexually Transmitted Infections* 87(2): 88–93. 20966458 10.1136/sti.2010.043372 PMC3272710

[7] Hallett TB, Singh K, Smith JA, White RG, Abu-Raddad LJ, et al (2008) Understanding the impact of male circumcision interventions on the spread of HIV in southern Africa. *PLoS ONE* 3(5): e2212. 18493593 10.1371/journal.pone.0002212 PMC2387228

[8] Nagelkerke NJD, Moses S, de Vlas SJ, Bailey RC (2007) Modeling the public health impact of male circumcision for HIV prevention in high prevalence areas in Africa. *BMC Infectious Diseases* 7(1): 16–30. 17355625 10.1186/1471-2334-7-16 PMC1832203

[9] Njeuhmeli E, Forsythe S, Reed J, Opuni M, Bollinger L, et al (2011) Voluntary medical male circumcision: modeling the impact and cost of expanding male circumcision for HIV prevention in eastern and southern Africa. *PLoS Medicine* 8(11): e1001132. 22140367 10.1371/journal.pmed.1001132 PMC3226464

[10] Baeten JM, Celum C, Coates TJ (2009) Male circumcision and HIV risks and benefits for women. *The Lancet* 374(9685): 182–184. 10.1016/S0140-6736(09)61311-8 19616704

[11] Auvert B, Sobngwi-Tambekou J, Cutler E, Nieuwoudt M, Lissouba P, et al (2009) Effect of male circumcision on prevalence of high-risk human papillomavirus in young men: Results of a randomized controlled trial conducted in Orange Farm, South Africa. *Journal of Infectious Diseases* 199(1): 14–19. 19086814 10.1086/595566 PMC2821597

[12] Gray RH, Serwadda D, Kong X, Makumbi F, Kigozi G, et al (2010) Male circumcision decreases acquisition and increases clearance of high risk human papillomavirus in HIV-negative men: a randomized trial in Rakai, Uganda. *Journal of Infectious Diseases* 201(10): 1455–1462. 20370483 10.1086/652184 PMC2882881

[13] Sobngwi-Tambekou J, Taljaard D, Lissouba P, Zara K, Puren A, et al (2009) Effect of HSV-2 serostatus on acquisition of HIV by young men: results of a longitudinal study in Orange Farm, South Africa. *Journal of Infectious Diseases* 199(7): 958–964. 19220143 10.1086/597208 PMC2868899

[14] Tobian AAR, Serwadda D, Quinn TC, Kigali G, Gravatt PE, et al (2009) Male circumcision for the prevention of HSV-2 and HPV infections and syphilis. *New England Journal of Medicine* 360(13): 1298–1308. 19321868 10.1056/NEJMoa0802556 PMC2676895

[15] Drain PK, Halperin DT, Hughes JP, Klausner JD, Bailey RC (2006) Male circumcision, religion, and infectious diseases: an ecologic analysis of 118 developing countries. *BMC Infectious Diseases* 6(1): 172–182. 17137513 10.1186/1471-2334-6-172 PMC1764746

[16] Gray RH, Wawer MJ, Serwadda D, Kigali G (2009) The role of male circumcision in the prevention of human papillomavirus and HIV infection. *Journal of Infectious Diseases* 199(1): 1–3. 19086812 10.1086/595568

[17] Nielson CM, Schiaffino MK, Dunne EF, Salemi JL, Giuliano AR (2009) Associations between male and anogenital human papillomavirus infection and circumcision by anatomic site sampled and lifetime number of female sex partners. *Journal of Infectious Diseases* 199(1): 7–13. 19086813 10.1086/595567 PMC5068969

[18] Wawer MJ, Tobian AAR, Kigozi G, Kong X, Gravitt PE, et al (2011) Effects of circumcision of HIV-negative men on transmission of human papillomavirus to HIV-negative women: a randomised trial in Rakai, Uganda. *The Lancet* 377(9761): 209–218. 10.1016/S0140-6736(10)61967-8 PMC3119044 21216000

[19] Agarwal SS, Sehgal A, Sardana S, Kumar A, Luthra UK. 1993. Role of male behaviour in cervical carcinogenesis among women with one lifetime sexual partner. *Cancer* 72(5): 166–169. 10.1002/1097-0142(19930901)72:5<1666::aid-cncr2820720528>3.0.co;2-m 8348498

[20] Castellsagué X, Bosch FX, Muñoz N, Meijer CJLM, Shah KV, et al (2002) Male circumcision, penile human papillomavirus infection, and cervical cancer in female partners. *New England Journal of Medicine* 346(15): 1105–1112. 11948269 10.1056/NEJMoa011688

[21] Mugo N, Dadabhai SS, Bunnell R, Williamson J, Bennett E, et al (2011) Prevalence of herpes simplex virus type 2 infection, human immunodeficiency virus/herpes simplex virus type 2 co-infection, and associated risk factors in a national, population-based survey in Kenya. *Sexually Transmitted Diseases* 38(11): 1059–1066. 21992985 10.1097/OLQ.0b013e31822e60b6

[22] Grosskurth H, Mosha F, Todd J, Mwijarubi E, Klokke E, et al (1995) Impact of improved treatment of sexually transmitted diseases on HIV infections in rural Tanzania: randomised controlled trial. *The Lancet* 346(8974): 530–536. 10.1016/s0140-6736(95)91380-7 7658778

[23] White RG, Orroth KK, Glynn JR, Freeman EE, Bakker RJ, et al (2008) Treating curable sexually transmitted infections to prevent HIV in Africa: still an effective control strategy? *Journal of Acquired Immune Deficiency Syndromes* 47(3): 346–353. 18176323 10.1097/QAI.0b013e318160d56a PMC3776949

[24] Feuer C. (2010) A cautious nod to the cut. Women weigh in on medical male circumcision. *ALQ/Mujeres AIDS Legal Network* July.

[25] Gostin LO, Hankins CA (2008) Male circumcision as an HIV prevention strategy in sub-Saharan Africa. *JAMA* 300(21): 2539–2541. 19050196 10.1001/jama.2008.752

[26] Hankins C (2007) Male circumcision: Implication for women as sexual partners and parents. *Reproductive Health Matters* 15(29): 62–67. 17512377 10.1016/S0968-8080(07)29311-5

[27] Wawer MJ, Makumbi F, Kigozi G, Serwadda D, Watya S, et al (2009) Circumcision in HIV-infected men and its effect on HIV transmission to female partners in Rakai, Uganda: a randomised controlled trial. *Lancet* 374(2685): 229–237. 19616720 10.1016/S0140-6736(09)60998-3 PMC2905212

[28] Gray RH, Kiwanuka N, Quinn TC, Sewankambo NK, Serwadda D, et al (2000) Male circumcision and HIV acquisition and transmission: cohort studies in Rakai, Uganda. *AIDS* 14(15): 2371–2381. 11089626 10.1097/00002030-200010200-00019

[29] Lagarde E, Taljaard D, Adrian P, Rain-Taljaard R, Auvert B (2003) Acceptability of male circumcision as a tool for preventing HIV infection in a highly infected community in South Africa. *AIDS* 17(1): 89–95. 12478073 10.1097/00002030-200301030-00012

[30] Rain-Taljaard RC, Lagarde E, Taljaard DJ, Campbell C, MacPhail C, et al (2003) Potential for an intervention based on male circumcision in a South African town with high levels of HIV infection. *AIDS Care* 15(3): 315–327. 12828151 10.1080/0954012031000105379

[31] Westercamp N, Bailey RC (2007) Acceptability of male circumcision for prevention of HIV/AIDS in sub-Saharan Africa: a review. *AIDS Behavior* 11(3): 341–355. 17053855 10.1007/s10461-006-9169-4 PMC1847541

[32] Obure AFXO, Nyambedha EO, Oindo BO (2011) Interpersonal influences in the scale-up of male circumcision services in a traditionally non-circumcising community in rural western Kenya. *Global Journal of Community Psychology Practice* 1(3): 1–11.

[33] Riess TH, Achieng' MM, Otieno S, Ndinya-Achola JO, Bailey RC (2010) “When I was circumcised I was taught certain things”: risk compensation and protective sexual behavior among circumcised men in Kisumu, Kenya. *PLoS ONE* 5(8): e12366. 20811622 10.1371/journal.pone.0012366 PMC2928269

[34] Layer EH, Beckham SW, Mgeni L, Shembilu C, Momburi RB, et al (2013) “After my husband's circumcision, I know that I am safe from diseases”: Women's attitudes and risk perceptions towards male circumcision in Iringa, Tanzania. *PLoS ONE* 8(8): e74391 Doi:10.1371/journal.pone.0074391 24009771 10.1371/journal.pone.0074391 PMC3756960

[35] National AIDS and STI Control Programme, Ministry of Health, Kenya. (2013) Kenya AIDS Indicator Survey 2012 Preliminary Report. Nairobi, Kenya, September.

[36] Kenya National Bureau of Statistics and ICF Macro. (2010) Kenya Demographic and Health Survey 2008–2009. Calverton, Maryland.

[37] Miles MB, Huberman AM. (1994) *Qualitative data analysis*. Thousand Oaks: Sage.

[38] Patton M. (2002) *Qualitative research and evaluation methods*. Thousand Oaks: Sage.

[39] ATLAS.ti. Version 5.2. [computer software] (2008) Berlin, Scientific Software Development.

[40] Strauss A, Corbin J. (1998) Basics of qualitative research techniques and procedures for developing grounded theory (2nd edition). Thousand Oaks: Sage.

[41] Glaser BG, Strauss AL. (1967) The discovery of grounded theory: strategies for qualitative research. Chicago: Aldine.

[42] Boeije H. (2002) A purposeful approach to the constant comparative method in the analysis of qualitative interviews. *Quality & Quantity* 36(4), 391–409.

[43] Bailey RC, Muga R, Poulussen R, Abicht H (2002) The acceptability of male circumcision to reduce HIV infections in Nyanza Province, Kenya. *AIDS Care* 14(1): 27–40. 11798403 10.1080/09540120220097919

[44] Mattson CL, Bailey RC, Muga R, Poulussen R, Onyango T (2005) Acceptability of male circumcision and predictors of circumcision preference among men and women in Nyanza Province, Kenya. *AIDS Care* 17(2): 182–194. 15763713 10.1080/09540120512331325671

[45] Kigozi G, Lukabwe I, Kagaayi J, Wawer MJ, Nantume B, et al (2009) Sexual satisfaction of women partners of circumcised men in a randomized trial of male circumcision in Rakai, Uganda. *BJU International* 104(11): 1698–1701. 19522862 10.1111/j.1464-410X.2009.08683.x

[46] Krieger JN, Mehta SD, Bailey RC, Agot K, Ndinya-Achola JO, et al (2008) Adult male circumcision: effects on sexual function and sexual satisfaction in Kisumu, Kenya. *The Journal of Sexual Medicine* 5(11): 2610–2622. 18761593 10.1111/j.1743-6109.2008.00979.x PMC3042320

[47] Waldinger MD, Quinn P, Dilleen M, Mundayat R, Schweitzer DH, et al (2005) A multinational population survey of intravaginal ejaculation latency time. *Journal of Sexual Medicine* 2(4): 492–497. 16422843 10.1111/j.1743-6109.2005.00070.x

[48] Waldinger MD, McIntosh J, Schweitzer DH (2009) A five-nation survey to assess the distribution of the intravaginal ejaculatory latency time among the general male population. *Journal of Sexual Medicine* 6(10): 2888–2895. 19627471 10.1111/j.1743-6109.2009.01392.x

[49] Cassell MM, Halperin DT, Shelton JD, Stanton D (2006) Risk compensation: the Achilles' heel of innovations in HIV prevention? *British Medical Journal* 332(7541): 605–607. 16528088 10.1136/bmj.332.7541.605 PMC1397752

[50] Herman-Roloff A, Bailey RC, Agot K. (2011) Factors associated with the early resumption of sexual activity following medical male circumcision in Nyanza Province, Kenya. Poster presented at the 16th International Conference on AIDS and STIs in Africa, 06 December, in Addis Ababa, Ethiopia. 10.1007/s10461-011-0073-1 PMC3677080 22052231

[51] Odoyo-June E, Rogers JH, Jaoko W, Bailey RC (2013) Factors associated with resumption of sex before complete wound healing in circumcised HIV-positive and HIV-negative men in Kisumu, Kenya. *Journal of Acquired Immune Deficiency Syndromes* 62(4): 465–470. 23242159 10.1097/QAI.0b013e3182800710 PMC3796143

[52] Westercamp M, Bailey RC, Bukusi EA, Montandon M, Kwena K, et al (2010) Male circumcision in the general population of Kisumu, Kenya: beliefs about protection, risk behaviors, HIV, and STIs. *PLoS ONE* 5(12): e15552. 21179493 10.1371/journal.pone.0015552 PMC3002946

[53] Bailey RC, Muga R, Ondiege M, Poulussen R. (1999) Acceptability of male circumcision as a strategy to reduce STD/HIV infections among the Luo in western Kenya, abstract #503. In: *Abstract Guide: 13th Meeting of the International Society for Sexually Transmitted Diseases Research*, p. 274. Presented at the 13th Meeting of the International Society for Sexually Transmitted Diseases Research, July 11–14 in Denver, USA.

